# Malaria in Pregnancy

**DOI:** 10.1155/S1064744997000100

**Published:** 1997

**Authors:** Lisa M. Hollier, April L. Ericksen, Susan M. Cox

**Affiliations:** Department of Obstetrics and Gynecology University of Texas Southwestern Medical Center 5323 Harry Hines Boulevard Dallas TX USA

## Abstract

This review summarizes the epidemiology, clinical course, and diagnosis of malaria. The influence of infection during pregnancy upon maternal and neonatal anemia, stillbirth, preterm labor, low birth weight, and congenital malaria is discussed. Options for treatment and prophylaxis during pregnancy are presented.


Infectious Diseases in Obstetrics and Gynecology 5:45–51 (1997)
© 1997 Wiley-Liss, Inc.

### Malaria in Pregnancy

**Lisa M. Hollier,* April L. Ericksen, and Susan M. Cox**
*Department of Obstetrics and Gynecology, University of Texas Southwestern Medical Center, Dallas, TX*

ABSTRACT

**This review summarizes the epidemiology, clinical course, and diagnosis of malaria. The influence of infection during pregnancy upon maternal and neonatal anemia, stillbirth, preterm labor, low birth weight, and congenital malaria is discussed. Options for treatment and prophylaxis during pregnancy are presented. Infect. Dis. Obstet. Gynecol. 5:45–51, 1997.** © 1997 Wiley-Liss, Inc.

KEY WORDS
*Plasmodium;* anemia; fever; fetus

While malaria is generally considered a disease of the third world, 1,419 cases were reported in the United States last year.[1] Because the disease is rare, practitioners may be unaware of the presentation. Malaria is a potentially fatal illness if not diagnosed and treated in a timely fashion.

### PATHOGENESIS

Malaria is caused by the genus *Plasmodium.* There are four species which infect man: *P. falciparum, P. vivax, P. ovale,* and *P. malariae.*[2] The infection is transmitted through the *Anopheles* mosquito. The life cycle is complex with both sexual and asexual reproduction (Fig. 1). The mosquito injects plasmodial sporozoites from its salivary glands into the bloodstream of its victim. The sporozoites in turn infect hepatocytes where they develop and multiply in a process known as extra-erythrocytic schizogony. Within 1–2 weeks, the hepatocytes rupture releasing merozoites. These merozoites invade red blood cells and begin erythrocyte schizogony—maturation through the trophozoite stage to become schizonts. Schizonts then develop into mature merozoites which are released from red blood cells to repeat the cycle. Some of the merozoites will develop into male and female gametocytes. When the female *Anopheles* mosquito ingests the gametocytes, sexual reproduction occurs within the mosquito producing additional sporozoites.[2] *P. vivax* and *P. ovale* have a dormant phase. Rather than entering extra-erythrocytic schizogony, the sporozoites become hypnozoites. In this form, they do not cause symptoms, however, they may reactivate at a later time causing clinical illness.

*P. falciparum* is the most widely distributed of the malaria species. It is found throughout Mexico, Haiti, and the Dominican Republic. It is also found in the equatorial regions of South America, Africa, and Asia. *P. vivax* is found in the Americas, Middle East, and North Africa.

The species *P. falciparum* is of special concern for three reasons. First, it is widely distributed. Second, strains of *P. falciparum* continue to emerge which are resistant to chloroquine. Finally, *P. falciparum* causes the most serious complications because it results in higher levels of parasitemia.[3]

### EPIDEMIOLOGY

The incidence of malaria is increased during pregnancy.[3] In holoendemic areas, the peak prevalence of *P. falciparum* parasitemia occurs between 9 and 16 weeks' gestation.[4] Primigravidas in particular

*Correspondence to: Dr. Lisa M. Hollier, Department of Obstetrics and Gynecology, University of Texas Southwestern Medical Center, 5323 Harry Hines Boulevard, Dallas, TX 75235-9032.

Received 10 October 1996

Accepted 9 May 1997

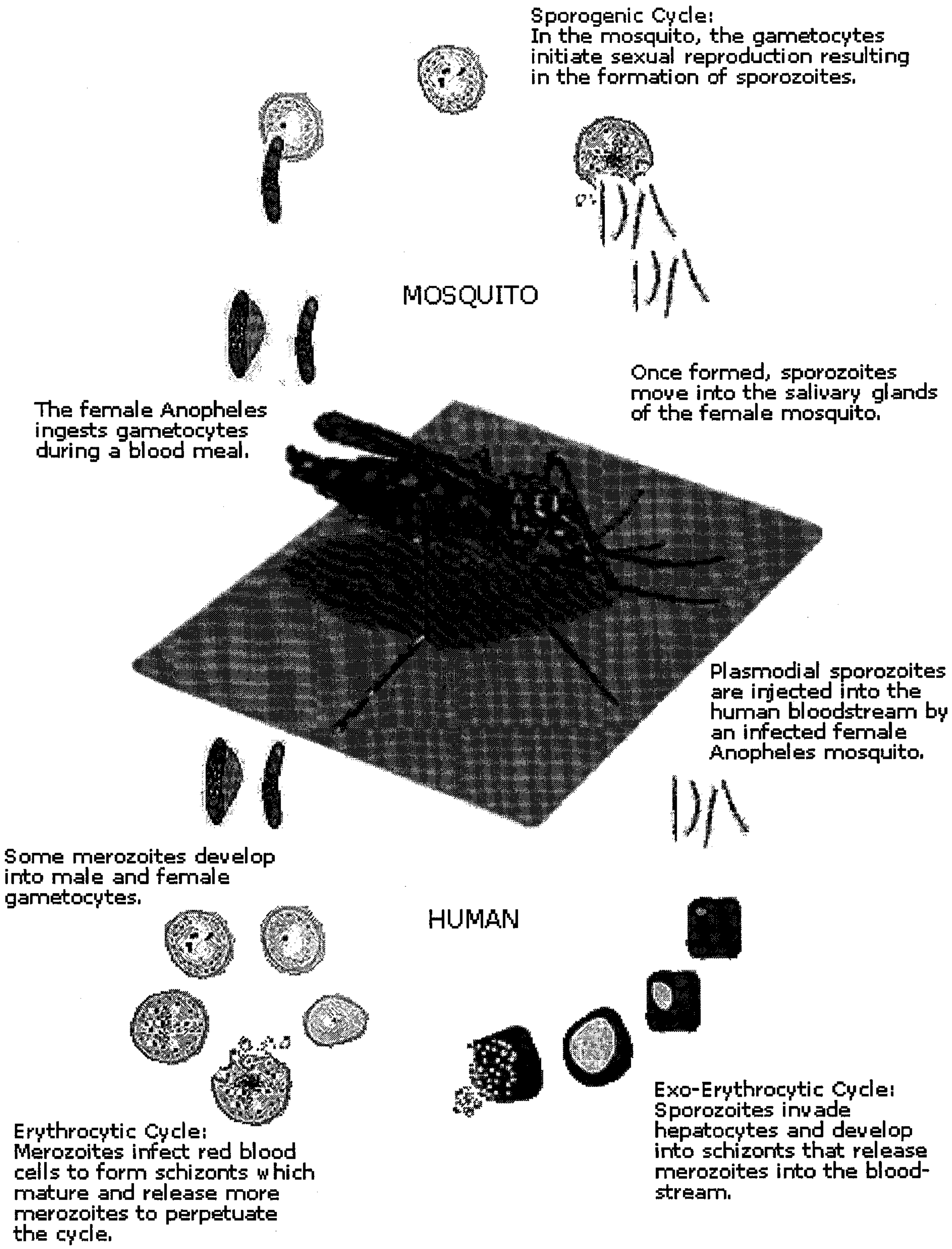

Fig. 1. *Plasmodium* life cycle (adapted from Zucker and Campbell[2]).

have increased susceptibility to malaria when compared to multigravid women. This vulnerability occurs even in areas where malaria is endemic.[5]

The reason for the increased susceptibility in pregnancy has long been a topic of research. Postulated mechanisms include alterations in hormonal factors as well as changes in cell-mediated and humoral antibody responses. Recently, several investigators have found that a placental protein, chondroitin sulfate A (CSA), may play a role.[6,7] Malaria-infected red blood cells produce surface proteins that allow them to bind to particular tissues. Fried and Duffy[8] have isolated red blood cells infected with *P. falciparum* from human placentas. These cells bind only to CSA and not to other known infected red blood cell receptors. These cells also bind to uninfected placentas in the same distribution as in naturally infected placentas. The authors propose that a woman becomes highly susceptible to malarial infection during her initial pregnancy when she first provides the placental substrate CSA. This exposure selects CSA-binding parasites for growth. With subsequent pregnancies, the woman develops increasing immunity to the selected subpopulation, reducing the frequency and severity of infection.

### COMPLICATIONS OF MALARIA IN PREGNANCY

A wide variety of complications can arise from malarial infection. Anemia is very common and occurs even in endemic regions. Abortion and premature delivery can occur in women without immunity. Intrauterine growth restriction, congenital malaria, and perinatal death are also risks.

### Anemia

The prevalence of anemia is highest between 16 and 28 weeks' gestation, following the peak prevalence of parasitemia.[9,10] Non-immune women will develop significant anemia with malarial infection.

The mechanism for the anemia is multifaceted. Immune-mediated hemolysis occurs in the peripheral circulation. In addition, red cells coated with immune complexes are cleared from the circulation by the spleen. Sequestration of infected erythrocytes in the spleen, liver, bone marrow, and placenta also decreases the hematocrit. The degree of splenomegaly has been correlated with the severity of anemia in a study by Brabin et al.[3]

Nutritional deficiencies further compound the anemia. Iron stores may be decreased by repeated pregnancies as well as inadequate diet. Folate deficiency with resulting megaloblasts occurs when the diet is unable to supply the increased demands of erythropoiesis.

Dyserythropoiesis is yet another potential contributor to the anemia observed in infected patients. This abnormal bone marrow response to anemia has been observed in children[11] and includes erythroblast multinuclearity, karyorrhexis, incomplete and unequal amitotic nuclear division, and cytoplasmic bridging.

In addition to maternal anemia, malaria is also associated with fetal anemia. Brabin[12] compared maternal and cord hemoglobin values in malaria-endemic and non-endemic regions. The range of maternal values in the population of pregnant women not exposed to malaria was 4.7–14.2 g/dl. The lowest cord value in this series of reports was 12.6 g/dl for a severely iron-deficient group of women (Hgb 4.7 g/dl). In contrast, in pregnant populations from malaria-endemic regions, cord hemoglobin values less than 13 were not uncommon and values as low as 7.4 g/dl were found in certain populations. Thus, it appears that exposure to malaria produces a degree of fetal anemia which seems greater than would be expected as a result of maternal iron deficiency alone.

### Stillbirth

Malaria is associated with an increased risk of stillbirth. While data to determine the precise mechanism of fetal death are lacking, several risk factors have been identified. Primigravidity is associated with stillbirth rates in excess of 10% in rural Gambia where malaria is endemic. For multigravidas, rates range from 0.9 to 6.9% in malaria-endemic areas. Other associated factors are hyperpyrexia,[14] severe anemia,[13] placental parasitemia, and hypoglycemia.[14] When placental infection occurs early in gestation, spontaneous abortion can result.

### Preterm Labor

There is currently little data demonstrating a causal relationship between malaria and preterm labor. Studies from sub-Saharan Africa are often limited by imprecise estimates of gestational age.

### Low Birth Weight

The prevalence of low birth weight (<2,500 g) infants in malaria-endemic areas ranges from 15 to

30%.[17,18] Diverse factors are associated with the delivery of these small infants including environmental and demographic characteristics. Several studies have demonstrated that the prevalence of low birth weight infants is higher among primigravidas than multigravidas.[17,19]

Maternal complications of *Plasmodium* infection such as anemia are also associated with low birth weight. Both the multifactorial nature of the problem and difficulty in accurate assessment of gestational age make determination of the direct effect of malaria on birth weight elusive.

Contributions to abnormal growth by circulating malaria parasites, malaria-associated placental lesions, and maternal anemia were evaluated by Meuris and colleagues.[17] The prevalence of low birth weight was 15% in the total population; however, in women without these factors, only 6.4% of infants had low birth weight. When both circulating parasites and placental lesions were found at birth, the percentage of low birth weight was 25.9%, and rose to 29.2% when maternal anemia (Hgb < 10) was also present.

One theoretical explanation for the association between infection and fetal growth abnormalities is placental damage. Malaria infection causes a thickening of the trophoblastic basement membrane.[20–22] Placental sinusoids are closely packed with parasitized red blood cells. This, together with an excess of intervillous macrophages and perivillous fibrin deposition, may be expected to cause microcirculatory obstruction and a reduction in nutrients supplied to the fetus.

### Congenital Malaria

Congenital malaria is defined as clinical malaria with peripheral parasitemia within 2 weeks of birth. It is important to note that this infection can be acquired by the fetus during the pregnancy (congenital) or during labor (perinatally).[23] The incidence of congenital malaria in infants of immune mothers in endemic areas is 0.3%, compared with 1–10% in infants of non-immune mothers in the same area.[14]

Symptoms include anorexia, irritability, nausea, vomiting, and jaundice.[23] Splenomegaly and associated thrombocytopenia are very common. The characteristics of congenital malaria can be modified by maternal antibody status. Transplacental passage of maternal immunoglobulin in the third trimester can potentially prevent or result in milder or delayed disease in the neonate. The gradual disappearance of the antibodies allows for the acquisition of mosquito-transmitted disease by the infant in endemic areas.

### PRESENTATION AND DIAGNOSIS

Moore et al.[24] reviewed 59 cases of malaria in Texas that occurred from 1990 to 1993. Six of the patients in this series were pregnant. Fever was a universal finding and chills occurred in 96%. Headache was present in 86%. Other associated symptoms included nausea, vomiting, abdominal pain, diarrhea, and cough. Physical examination revealed splenomegaly in 40%. Laboratory abnormalities included elevated lactate dehydrogenase (>200 U/l), elevated bilirubin (>1.0 mg/dl), thrombocytopenia ($<150 \times 10^9$ l), elevated creatinine (>1 mg/dl), and anemia (Hct < 33 vol%).

Cerebral dysfunction is the most common severe manifestation of (*P. falciparum*) malaria in man. Symptoms can be gradual in onset or coma can follow a generalized convulsion. Other symptoms include delirium and focal "fits" without loss of consciousness.[25] Several hypotheses have been put forth to explain the disease process including sludging or obstruction of the cerebral vessels, as well as a breakdown of the vascular endothelium leading to cerebral edema.

Other complications of severe malaria infection include renal failure, adult respiratory distress syndrome, and intravascular hemolysis. *P. falciparum* is usually the etiologic agent; other species rarely cause severe disease.

Fever and chills occur at the time of rupture of erythrocytes containing merozoites.[26] Cytokines, e.g., tumor necrosis factor-alpha (TNF-$\alpha$) and interleukin-1, are both thought to play a role in the systemic manifestations of the illness. Cytoadherence also plays a role in the establishment of severe disease.

Both acute infection with malaria and its therapy are associated with hypoglycemia.[15,16] Pregnancy is associated with an increased sensitivity of the pancreatic $\beta$ cells to stimulants for insulin release. Therefore, pregnant patients are particularly susceptible to hyperinsulinemia caused by quinine and quinidine.

### Diagnosis

A fresh sample of blood should be obtained for both thin and thick smear preparations. For thin smears, Wright stains alone are not satisfactory; the addition of 3% Giemsa or Giemsa alone improves the parasite yield. In inexperienced hands, the thin smear may be easier to read. The thick smear, however, provides the experienced technician higher sensitivity due to higher concentration of the parasites. Several smears may be necessary to establish the diagnosis in women with low parasite density.[2]

### TREATMENT

There are three basic principles which guide therapy: the infecting species, parasite density, and clinical status of the patient.[2] Early identification of the species is important because the illness caused by *P. falciparum* can progress rapidly. Pregnant women are at especially high risk; therefore, early and aggressive therapy is critical. In addition to giving a drug which rapidly kills the parasites, supportive therapy is very important. Fluid status and serum glucose need to be monitored. Daily blood smears should be obtained until negative.

Infections caused by *P. vivax, P. ovale,* and *P. malariae* should be treated with chloroquine. *P. vivax* and *P. ovale* both have dormant forms, referred to as hypnozoites, which are asymptomatic. These forms are not susceptible to chloroquine and, therefore, in non-pregnant individuals a course of primaquine is added at the completion of the initial therapy. Prior to treatment with primaquine, individuals at risk for glucose-6-phosphate deficiency should be evaluated. Those patients with <10% residual enzyme activity should not be treated due to the risk of hemolytic disease.

Primaquine can cause hemolytic disease in the fetus, so it is *not* recommended in pregnancy. Rather, the gravida should be maintained on prophylactic doses of chloroquine until delivery in an effort to prevent relapse during gestation. She can then be treated with primaquine in the postpartum period.[2]

When infection is caused by *P. falciparum,* it is important to know if the infection was acquired in an area with reported chloroquine resistance. If the infection was acquired in an area without reported resistance, treatment with oral chloroquine is adequate. For infections thought to be caused by resistant organisms, quinine and clindamycin can be used. Mefloquine is another alternative that can be used in pregnancy (Table 1). Little information is currently available on the use of artemisinin derivatives in pregnancy, however, their use is recommended for the treatment of mefloquine-resistant, *P. falciparum* infections after the first trimester.[27]

**TABLE 1. Treatment options safe for use during pregnancy**

| Drug | Dosage |
|---|---|
| CQ sensitive | |
| Chloroquine | 10 mg base/kg p.o. followed by 10 mg base/kg p.o. at 24 h and 5 mg base/kg p.o. at 48 h |
| CQ resistant | |
| Quinine sulfate | 650 mg salt p.o. q 8 h × 3–7 days |
| with clindamycin | 450 mg p.o. q 8 h × 3 days |
| Mefloquine | 15 mg base/kg in a single dose |
| Intravenous | |
| Quinidine gluconate | 10 mg/kg loading dose over 1–2 h<br>0.02 mg/kg/min continuous maintenance infusion |

[a]CQ = chloroquine prophylaxis.
[b]Antimalarial drug doses can be expressed in terms of either the base or the salt of the drug. Because dosages differ for the different forms, clinicians must specify the form desired.

Indications for intravenous therapy include inability to tolerate oral therapy and parasite density exceeding 5%.[2] Intravenous quinidine is given to maintain levels of 3–7 mg/l (Table 1). These patients require central venous pressure monitoring as well as continuous cardiac monitoring for early detection of prolongation of the QT interval. The 3-day course of therapy can be completed orally when the parasite density falls below 1% and the patient can tolerate oral medication. A 7-day course is needed if the disease is acquired in Thailand.

Potential indications for exchange transfusion include parasite density >10% or severe complications such as altered mental status, non-volume overload pulmonary edema, or renal disease. Intravenous quinidine is administered during the exchange.[2]

### PREVENTION AND CHEMOPROPHYLAXIS

### Prevention

Basic principles to improve the control of malaria include 1) protection of the human from the *Anopheles* mosquito, 2) reduction of the breeding and survival of the mosquito, 3) aggressive and early treat-

**TABLE 2. Prophylactic regimens safe for use in pregnancy**

| Drug | Dosage |
|---|---|
| Chloroquine | 300 mg base p.o. q week |
| Proguanil | 200 mg p.o. daily (may be combined with chloroquine) |
| Pyrimethamine/ sulfadoxine | 75 mg/1,500 mg p.o. single dose, repeat at beginning of the third trimester |
| Mefloquine | 250 mg salt p.o. q week |

ment of the human disease, and 4) establishment of surveillance and education programs.[28]

Two newer devices for human protection include insecticide-impregnated bed nets and curtains.[29] At the present time, studies of their effectiveness are small and financial factors may limit their usefulness.

### Prophylaxis

Because prophylactic treatment can fail, it should be emphasized that the optimal method for preventing malarial infection in the non-immune gravida is to refrain from travel to malaria-endemic regions. When such travel is unavoidable, however, there are several prophylactic regimens safe for use in pregnancy (Table 2). The cost-effectiveness of these regimens for women living in endemic regions remains to be proven.

Mefloquine has been evaluated as prophylaxis for chloroquine-resistant malaria.[30] In a double-blind, placebo-controlled study in Thailand, mefloquine gave ≥86% protection against *P. falciparum* and complete protection against *P. vivax*. There was no difference in either the mean birth weight or the mean gestational age at delivery between the two groups. Mefloquine is not recommended for use in the first trimester or in individuals with known neurologic or psychiatric disorders. The drug may provoke severe neuropsychiatric reactions.[31]

In Malawi, another area with chloroquine-resistant malaria, prophylaxis was evaluated in primi- and secundagravidas.[32] Three regimens were compared: chloroquine treatment followed by chloroquine prophylaxis (CQ/CQ), sulfadoxine/pyrimethamine treatment followed by chloroquine prophylaxis (SP/CQ), and sulfadoxine/pyrimethamine treatment and prophylaxis (SP/SP). Combined treatment and prophylaxis with sulfadoxine/pyrimethamine resulted in a marked decrease in maternal parasitemia (32% for CQ/CQ and 14% for SP/CQ vs 3% for SP/SP). Placental parasitemia was also significantly reduced (32%, 25%, and 9%, respectively, for CQ/CQ, SP/CQ, and SP/SP). Three percent of SP-treated infants developed scleral icterus; all cases resolved without complications after phototherapy.

The effect of proguanil, either alone or in combination with chloroquine, was evaluated by Mutabingwa et al.[33] Therapy was noted to reduce the rate of low birth weight infants, however, due to the small numbers of participants, the differences were not significant.[33] Placental parasitemia was significantly reduced. In primigravidas, the prevalence of severe anemia was also reduced.

### SUMMARY

Malaria is caused by four species of *Plasmodium*. Infections in pregnant women are associated with multiple complications including anemia, low birth weight, and stillbirth. A high index of suspicion for malaria in women emigrating from or traveling through endemic areas is vital. Prompt diagnosis and initiation of appropriate therapy may be lifesaving.

### REFERENCES

1. Centers for Disease Control: Final 1995 reports of notifiable diseases. MMWR 45:742, 749–754, 1996.
2. Zucker JR, Campbell CC: Malaria. Principles of prevention and treatment. Infect Dis Clin North Am 7:547–567, 1993.
3. Brabin BJ, Ginny M, Sapau J, Galme K, Paino J: Consequences of maternal anaemia and outcome of pregnancy in a malaria endemic area in Papua New Guinea. Ann Trop Med Parasitol 84:11–24, 1990.
4. Brabin BJ, Ginny M, Alpers M, Brabin L, Eggelte T, Van der Kaay HJ: Failure of chloroquine prophylaxis for falciparum malaria in pregnant women in Madang, Papua New Guinea. Ann Trop Med Parasitol 84:1–9, 1990.
5. Fleming AF, Ghatoura GBS, Harrison KA, Briggs ND, Dunn DT: The prevention of anaemia in pregnancy in primigravidae in the Guinea Savanna of Nigeria. Ann Trop Med Parasitol 80:211–233, 1986.
6. Rogerson SJ, Chiayaroj SC, Ng K, Reeder JC, Brown GV: Chondroitin sulfate A is a cell surface receptor for *Plasmodium falciparum*-infected erythrocytes. J Exp Med 182:15–20, 1995.
7. Robert C, Pouvelle B, Meyer P, et al.: Chondroitin-4-sulfate (proteoglycan), a receptor for *Plasmodium falciparum*-infected erythrocyte adherence on brain microvascular endothelial cells. Res Immunol 146:383–393, 1995.

8. Fried M, Duffy PE: Adherence of *Plasmodium falciparum* to chondroitin sulfate A in the human placenta. Science 272:1502–1504, 1996.
9. McGregor IA: Malaria—Recollections and observations. Trans R Soc Trop Med Hyg 78:1–8, 1984.
10. Brabin BJ: An analysis of malaria in pregnancy in Africa. Bull WHO 61:1005–1016, 1983.
11. Abdalla S, Weatherall DJ, Wickramasinghe SN, Hughes M: The anemia of *P. falciparum* malaria. Br J Haematol 46:171–183, 1986.
12. Brabin B: Fetal anaemia in malarious areas: Its causes and significance. Ann Trop Paediatr 12:303–310, 1992.
13. Mutabingwa TK: Malaria and pregnancy: Epidemiology, pathophysiology and control options. Acta Trop 57: 239–254, 1994.
14. Klufio CA: Malaria in pregnancy. Papua New Guinea Med J 35:249–257, 1992.
15. Looareesuwan S, Phillips RE, White NJ, et al.: Quinine and severe falciparum malaria in late pregnancy. Lancet 2:4–8, 1985.
16. Nathwani D, Currie PF, Douglas JG, Green ST, Smith NC: *Plasmodium falciparum* malaria in pregnancy: A review. Br J Obstet Gynaecol 99:118–121, 1992.
17. Meuris S, Piko BB, Eerens P, van Bellingham A, Dramaix M, Hennart P: Gestational malaria: Assessment of its consequences on fetal growth. Am J Trop Med Hyg 48:603–609, 1993.
18. Cot M, Lettesran JY, Miailhes P, Esveld M, Etya'ale D, Breart G: Increase of birthweight following chloroquine chemoprophylaxis during the first pregnancy: Results of a randomized trial in Cameroon. Am J Trop Med Hyg 53:581–585, 1995.
19. Morgan HG: Placental malaria and low birthweight neonates in urban Sierra Leone. Ann Trop Med Parasitol 88:575–580, 1994.
20. Galbraith RM, Page Faulk W, Galbraith GMP, Holbrook TW: The human materno-fetal relationship in malaria. I. Identification of pigment and parasites in the placenta. Trans R Soc Trop Med Hyg 74:52–60, 1980.
21. Galbraith RM, Fox H, Hsi B, Galbraith GMP, Bray RS, Page Faulk W: The human materno-fetal relationship in malaria. II. Histological, ultrastructural and immunopathological studies of the placenta. Trans R Soc Trop Med Hyg 74:61–72, 1980.
22. Walter P, Gavin Y, Blot P: Placental pathologic changes in malaria: A histologic and ultrastructural study. Am J Pathol 109:332–344, 1982.
23. Karpuch J, Eshel G, Livne M, Ephros M: Congenital malaria in Israel—A case report. Isr J Med Sci 30:289–291, 1994.
24. Moore TA, Tomayko JF, Wierman AM, Rensimer ER, White AC: Imported malaria in the 1990s. Arch Fam Med 3:130–136, 1994.
25. Warrell DA: Pathophysiology of severe falciparum malaria in man. Parasitology 94:S53–S76, 1987.
26. Miller LH, Good MF, Milon G: Malaria pathogenesis. Science 264:1878–1883, 1994.
27. World Health Organization: The role of artemisinin and its derivatives in the current treatment of malaria (1994–1995): Report of an informal consultation convened by WHO in Geneva, 27–29 September 1993. Geneva: World Health Organization, 1994.
28. Beaver PC, Jung RC: Animal Agents and Vectors of Human Disease. 5th ed. Philadelphia: Lea & Febiger, p 72, 1985.
29. World Health Organization: The use of impregnated bed nets and other materials for vector borne disease control. WHO/VBC/89.981. Geneva: World Health Organization, p 45, 1989.
30. Nosten F, ter Kuile F, Maelankiri L, et al.: Mefloquine prophylaxis prevents malaria during pregnancy: A double-blind, placebo-controlled study. J Infect Dis 169:595–603, 1994.
31. Bradley DJ, Warhurst DC: Malaria prophylaxis. Guidelines for travellers from Britain. BMJ 310:709–714, 1995.
32. Schultz LJ, Steketee RW, Macheso A, Kazembe P, Chitsulo L, Wirima JJ: The efficacy of anti-malarial regimens containing sulfadoxine-pyrimethamine and/or chloroquine in preventing peripheral and placental *Plasmodium falciparum* infection among pregnant women in Malawi. Am J Trop Med Hyg 51:515–522, 1994.
33. Mutabingwa TK, Malle LN, deGeus A, Dosting J: Malaria chemosuppression in pregnancy. I. Tropical Geog Med 45:6–14, 1993.
